# The complete mitochondrial genome of the New Zealand parasitic roundworm *Haemonchus contortus* (Trichostrongyloidea: Haemonchidae) field strain NZ_Hco_NP

**DOI:** 10.1080/23802359.2019.1624634

**Published:** 2019-07-12

**Authors:** Nikola Palevich, Paul Maclean, Abdul Baten, Richard Scott, David M. Leathwick

**Affiliations:** Grasslands Research Centre, AgResearch Ltd, Palmerston North, New Zealand

**Keywords:** *Haemonchus contortus*, roundworm, gastrointestinal, trichostrongyloid, nematode, phylogeny

## Abstract

The complete mitochondrial genome of the New Zealand parasitic nematode *Haemonchus contortus* field strain NZ_Hco_NP was sequenced and annotated. The 14,001 bp-long mitogenome contains 12 protein-coding genes (atp8 gene missing), two ribosomal RNAs, 22 transfer RNAs, and a 583 bp non-coding region. Phylogenetic analysis showed that *H. contortus* NZ_Hco_NP forms a monophyletic cluster with the remaining three Haemonchidae species, and further illustrates the high levels of diversity and gene flow among Trichostrongylidae.

The large and highly variable mitochondrial (mt) genomes of helminths (worms), including parasitic nematodes (roundworms), are ideal sources of molecular markers suitable for studying population genetic structures and evolution. *Haemonchus contortus* NZ_Hco_NP was selected for genome sequencing as a representative of an anthelmintic-susceptible NZ field strain of *H. contortus*. The specimen was collected from the Palmerston North area (40°21.3′ S, 175°36.7′ E), and is stored and available upon request from AgResearch Ltd., Grasslands Research Centre. High molecular weight genomic DNA was isolated from multiple *H. contortus* adult males using a modified phenol:chloroform protocol (Palevich et al. 2017, 2019). Illumina HiSeq2500 and Pacific Biosciences (PacBio) (Macrogen, Korea) platforms were used to amplify the entire mitochondrial genome sequence (BioProject ID: PRJNA517503, GenBank accession number: CP035799).

The mitogenome (14,001 bp) is standard in size and comparable to the McMaster and ISE strains (Jex et al. 2008; Laing et al. 2013). For example, all genes are transcribed in the same direction, there is a lack of the Atp8 gene, it contains 12 protein-coding genes (PCGs), two rRNAs, 22 tRNAs, and an AT-rich region (583 bp). All 12 PCGs use standard ATN/TAN start/stop codons, respectively. The studied genome has a high T content (44.5%) and a low C content (6.3%), resulting in a very strong A + T bias (78.9%) and in particular the AT-rich region (91%). Gene order, sizes and all common organization features are relatively conserved among the 41 nematode mitogenomes (usually 13.6–14.3 kb) (Hu et al. 2003; Hu and Gasser 2006; Palevich et al. 2018).

The phylogenetic position of *H. contortus* was estimated using maximum-likelihood, implemented in RAxML version 8.2.11 (1000 bootstrap replications) (Stamatakis 2014), and the Bayesian inference (BI), implemented in MrBayes version 3.2.6 (default settings, four MCMC chains, 6.34–106 generations) (Huelsenbeck and Ronquist 2001) approaches. Mitogenome sequences of all 36 available nematode species were retrieved from GenBank. Analyses were performed both on the entire nucleotide sequences of the complete mitogenomes and using only the concatenated mitochondrial PCGs and rRNA genes, producing identical dendrogram topologies (Figure 1). *Haemonchus contortus* formed a monophyletic cluster with the remaining Trichostrongylidae species, which then formed a sister clade with the Strongylidae family. Overall, the dendrogram topology is highly congruent with the previous results of Jex et al. (2008). In the pursuit of improving the phylogenetic resolution within the phylum Nematoda, future efforts should focus on the availability of more complete mitogenomes across all nematode species, and especially for different strains/isolates.

**Figure 1.**
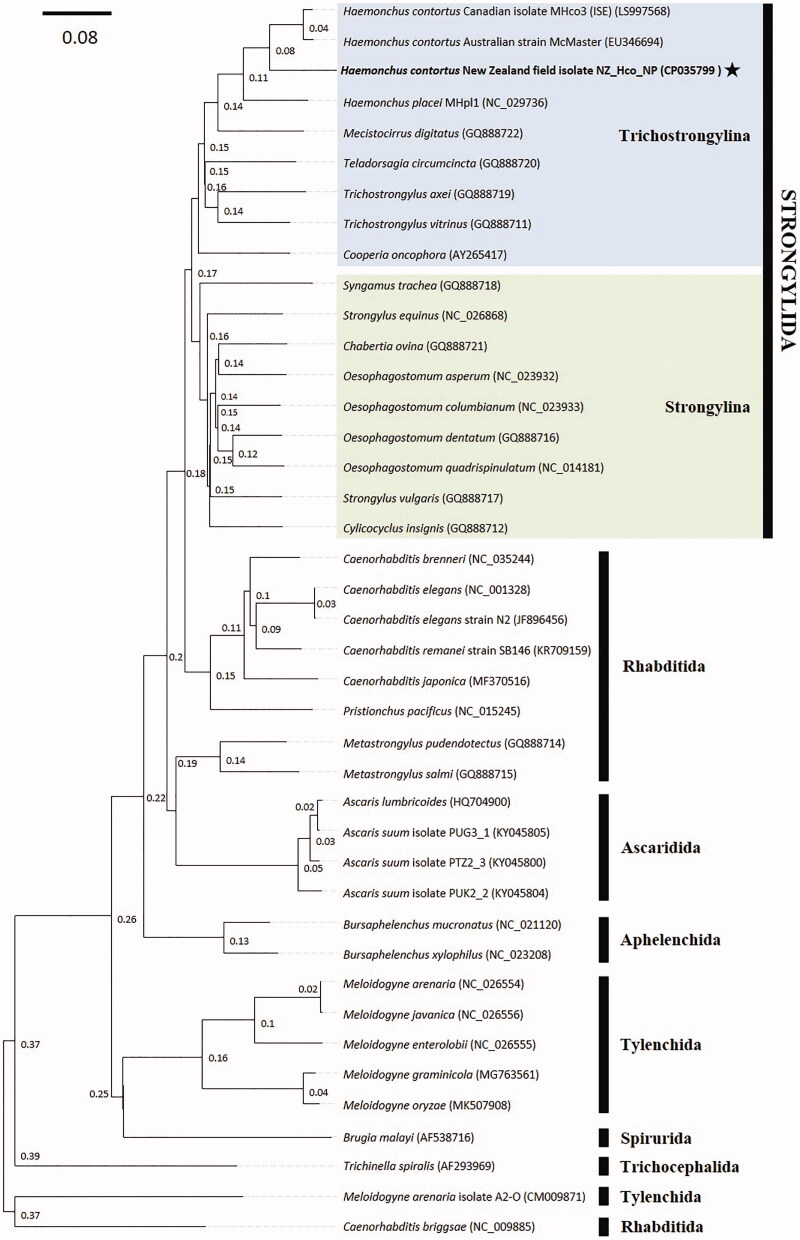
Phylogenetic analysis of the complete mt genomes for all 41 nematode species or isolates is available in GenBank. The evolutionary relationships between the *H. contortus* field strain NZ_Hco_NP (highlighted by a black star) and the two major suborders of the Strongylida are represented by coloured boxes (Trichostrongylina (blue) and Strongylina (green)). Species representing the Rhabditida, Ascaridida, Aphelenchida, Tylenchida, Spirurida, and Trichocephalida have been included as outgroups. Phylogenetic analysis was conducted using maximum-likelihood and Bayesian inference (MrBayes). The numbers above the midpoint of each tree branch represent the statistical support for each node (based on posterior probability score). The phylogram provided is presented to scale (scale bar = 0.08 estimated number of substitutions per site) and GenBank accession numbers are provided (in parentheses) for all reference sequences. An identical topology was found with maximum-likelihood; all nodes were supported by >99% bootstrap re-sampling (*n* = 1000).
